# Targeting phosphatases in cancer: suppression of many versus the ablation of one

**DOI:** 10.18632/oncotarget.27201

**Published:** 2019-11-12

**Authors:** Brandon M. D’Arcy, Aishwarya Prakash, Richard E. Honkanen

**Affiliations:** Department of Biochemistry and Molecular Biology, College of Medicine, University of South Alabama, Mobile, AL 36688, USA; Department of Biochemistry and Molecular Biology, College of Medicine, University of South Alabama, Mobile, AL 36688, USA

**Keywords:** serine/threonine phosphatases, LB-100, PPP2CA, PPP5C

LB-100 (3-(4-methylpiperazine-1-carbonyl)-7-oxabicyclo[2.2.1]heptane-2-carboxylic acid) is a promising antitumor drug currently in early stage clinical testing. LB-100 has cytotoxic activity against a variety of cancer cells in culture and marked anti-tumor activity in animal models of tumor development. Phase I clinical studies (NCT01837667) concluded that the safety parameters of LB-100 support continued development [1]. Phase Ib/II trials (NCT0388662 and NCT03027388), which will assess the safety and efficacy of LB-100 against myelodysplastic syndromes and recurrent glioblastomas (astrocytoma, glioblastoma multiforme, and giant cell glioblastoma) respectively, are ongoing.

**Figure 1:**
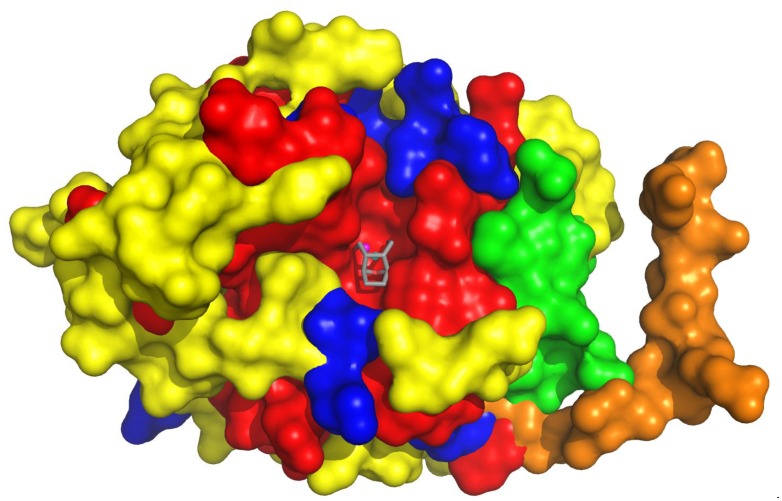
Superimposed surface representation of PP2AC and PPP5C with LB-100M/endothall positioned using the coordinates from the PPP5C:LB-100 complex. Red: residues that are conserved between PP2AC and PPP5C; blue: residues with similar physiochemical properties; yellow: residues with different physiochemical properties; green: conformation of residues unique to the crystal packing of PPP5C; orange: conformation of residues unique to the crystal packing of PP2AC; cyan: PPP5C metal ions; magenta: PP2AC metal ions; and grey: the 7-oxabicyclo[2.2.1]heptane-2,3-dicarbonyl moiety of LB-100. PDB ID 2IAE (PP2AC) [11] and 5WG8 (PPP5C:LB-100 complex) [2].

Early reports revealed that LB-100 acts as a catalytic inhibitor of serine/threonine phosphatase 2A (PP2AC: encoded by *PPP2CA/PPP2CB*). More recently, LB-100 was shown to also target PPP5C, inhibiting PP2AC and PPP5C with similar potency (IC_50_ = 0.4 and 1.8 μM for PP2AC and PPP5C, respectively) [2]. To determine the molecular basis for catalytic inhibition, we solved the high-resolution crystal structure of PPP5C in the presence of LB-100 at a resolution of 1.65 Å, which revealed clear electron density for the 7-oxabicyclo[2.2.1]heptane-2,3-dicarbonyl moiety, with the 2,3-dicarbonyl of LB-100 coordinating with both catalytic metals (M1 and M2). In order to accommodate the inhibitor, the side chains of Arg275 and Tyr451 adopt alternate conformations in relation to the active site metals, and the O7 bridgehead oxygen replaces the water molecule, which would otherwise coordinate with metal ion, M2. This causes the inhibitor to be tightly coordinated within the catalytic pocket, blocking substrate access to the catalytic metals (Figure 1). However, in our high-resolution structure [2], there is no density for the 3-(4-methyl-1-piperazine) ring, suggesting that either this region is flexible and does not contribute to binding, or that the methylpiperazine ring is labile in a biological setting. If the ring is released, then a metabolite (LB100M: 7-oxabicyclo[2.2.1]heptane-2,3-dicarboxylic acid; also called endothall) represents the biologically active compound. Assessment of LB100M levels in plasma and tumor tissue will be determined shortly, as they are secondary outcomes in an ongoing Phase II trial (NCT03027388).

The preclinical success of LB-100 argues that the PPP-family of phosphatases can indeed be targeted for the development of new antitumor drugs. However, it also brings up the question of which phosphatase(s) should be targeted. In addition to LB-100, other PPP-family inhibitors with antitumor activity include fostriecin, tautomycetin, tautomycin, and cantharidin [3]. All of these inhibitors are natural products, and they each inhibit PP2AC. However, to many, targeting PP2AC appears counterintuitive in the respect that several studies indicate PP2AC acts as a tumor suppressor. The role of PP2AC as a tumor suppressor originates from studies revealing that both Simian Virus 40 small T antigen and murine polyomavirus middle T antigen inhibit PP2AC, as part of the process by which they transform epithelial cells [4]. More recently, proteins known to have oncogenic activity in solid tumors (*i.e.* SET/I2PP2A and CIP2A) were shown to act as natural suppressors of PP2AC activity [5]. These observations argue that increasing, not inhibiting PP2AC activity, could circumvent tumor formation or progression. A counterpoint to this argument is that the genetic disruption of either *PPP2CA* or *PPP2CB* expression in mice results in early embryonic lethality. This observation indirectly supports the concept that inhibition of PPP2CA/B could have cytotoxic activity against cancer cells.

The aforementioned natural compounds also inhibit PPP1C and PPP5C, with fostriecin representing the only compound with marked selectivity for PP2AC. Another point to consider is that PP2AC represents a catalytic subunit that is shared with at least 18 different PPP2A holoenzymes. Thus, the existing data are consistent with the concept that the simultaneous suppression of several PPP-family phosphatases, which are essential regulators of cell cycle progression, the DNA damage response (DDR), numerous checkpoint control mechanisms and mitosis, may collectively generate antitumor activity. For example PPP1- and PPP2-holoenzymes have been implicated in complex crosstalk, acting to counter the activity of key kinetochore associated kinases (*i.e*. Aurora B, Plk1, Mps1, Bub1 and Cdk1) that control the KMN network (a major outer kinetochore signaling hub) [6]. Notably, the activity of these PPPases have been linked to the regulation of centromeric cohesion, mitotic spindle assembly, and numerous phosphorylation dependent mechanisms and processes that are under the surveillance of the spindle assembly checkpoint (SAC) [7, 8]. In the presence of unattached or aberrantly tensioned kinetochores, the SAC triggers metaphase-anaphase arrest and if not resolved, apoptosis [9, 10]. Several studies have shown that suppressing the activity or expression of PPP1, PPP2A, PPP4, PPP5, or PPP6 elicit cell cycle arrest and ultimately apoptosis from mitotic catastrophe. Moreover, during the DDR, PPP1, PPP2A, and PPP5 inhibition have been linked to mechanisms that trigger growth arrest or induce apoptosis.

Based on the reported literature, the antitumor actions of LB-100 are most apparent when used in combination with therapies associated with DDR-/genomic stress-induced apoptosis (*e.g.* cisplatin, doxorubicin) or SAC-triggering mitotic poisons (*e.g.* paclitaxel, vinblastine). These observations support the concept that existing therapies that trigger apoptosis in cancer cells can be augmented by the simultaneous suppression of the many PPPases that act to suppress the onset of apoptosis in the DDR, SAC, and other checkpoint control mechanisms. Here, it is important to consider that suppression, without ablation of activity may be needed to achieve cancer cell selective death, because compounds (*i.e.* microcystin) that potently inhibit PPP1C, PP2AC, PPP4C, and PPP5C have marked systemic toxicity. At the concentration used in animal studies that appear achievable in humans based on the early phase I data, LB-100 inhibits both PP2AC and PPP5C. LB100M/endothall lacks selectivity, and if present, is likely suppressing the activity of all cantharidin-sensitive phosphatases (PPP1C, PP2AC, PPP5C, and PPP6C). Regardless of whether the biological effects of LB-100 treatment are the result of suppressing one or several PPP family members, the promising results from these early clinical trials should not be ignored.
